# Supplementary material for: The F-box protein FBXL-5 governs vitellogenesis and lipid homeostasis in C. elegans
Source: Front Cell Dev Biol. 2024 Jun 14;12:1389077. doi: 10.3389/fcell.2024.1389077 (PMC11211535; doi:10.3389/fcell.2024.1389077)
Supplement: Supplementary file 2 [file DataSheet1.PDF]

Supplementary Material for

The F-box protein FBXL-5 governs vitellogenesis and lipid homeostasis in *C. elegans*

Peter C. Breen, Kendall G. Kanakanui, Martin A. Newman, Robert H. Dowen

Contents **Page**

1. Supplemental Tables S1-S3 2
2. Supplemental Figures S1-S7 6
3. References 13

Strain	Genotype	Reference
N2	Wild-type	Brenner, 1974
DLS258	*lin-4(e912) II; fbxl-5(rhd43) V; mgIs70[*P*vit-3::GFP]*	This study
DLS260	*lin-4(e912) II; fbxl-5(rhd56) V; mgIs70[Pvit-3::GFP]*	This study
DLS316	*fbxl-5(rhd43) V*	This study
DLS327	*lin-4(e912) II; fbxl-5(rhd43) V; mgIs70[*P*vit-3::GFP]; rhdEx73[*P*vha-6::mCherry::his-58::SL2::fbxl-5* + P*myo-2::mCherry]*	This study
DLS330	*lin-4(e912) II; fbxl-5(rhd43) V; mgIs70[*P*vit-3::GFP]; rhdEx76[*P*col-10::mCherry::his-58::SL2::fbxl-5* + P*myo-2::mCherry]*	This study
DLS333	*lin-29(n333) II; fbxl-5(rhd43) V*	This study
DLS344	*rhdEx93[*P*fbxl-5::mCherry::unc-54 3'UTR* + P*myo-3::GFP]*	This study
DLS445	*mgIs70[*P*vit-3::GFP]; rhdIs2[*P*vha-6::mCherry::his-58::SL2::fbxl-5 + cb-unc-119(+)]*	This study
DLS447	*mgIs70[*P*vit-3::GFP]; rhdIs4[*P*vha-6::mCherry::his-58::SL2::fbxl-5 + cb-unc-119(+)]*	This study
DLS476	*rhdIs2[*P*vha-6::mCherry::his-58::SL2::fbxl-5 + cb-unc-119(+)]*	This study
DLS477	*rhdIs4[*P*vha-6::mCherry::his-58::SL2::fbxl-5 + cb-unc-119(+)]*	This study
DLS490	*rict-1(mg360) II*	This study
DLS491	*rict-1(mg360) II; fbxl-5(rhd43) V*	This study
DLS492	*lin-29(n333) II; fbxl-5(rhd43) V*	This study
DLS537	*rhdSi42[*P*vit-3::mCherry::unc-54 3'UTR + cb-unc-119(+)] II*	Torzone et al., 2023
DLS561	*rhdSi42[*P*vit-3::mCherry::unc-54 3'UTR + cb-unc-119(+)] lin-4(e912) II*	This study
DLS708	*cul-6(ok1614) IV; mgIs70[*P*vit-3::GFP]; rhdIs4[*P*vha-6::mCherry::his-58::SL2::fbxl-5 + cb-unc-119(+)]*	This study
DLS709	*skr-3(ok365) V; mgIs70[*P*vit-3::GFP]; rhdIs4[*P*vha-6::mCherry::his-58::SL2::fbxl-5 + cb-unc-119(+)]*	This study
DLS726	*cul-6(ok1614) IV; skr-3(ok365) V; mgIs70[*P*vit-3::GFP]; rhdIs4[*P*vha-6::mCherry::his-58::SL2::fbxl-5 + cb-unc-119(+)]*	This study
DLS806	*cul-6(ok1614) IV; skr-3(ok365) skr-5(rhd269) skr-4(rhd283[W67*]) V; mgIs70[*P*vit-3::GFP]; rhdIs4[*P*vha-6::mCherry::his-58::SL2::fbxl-5 + cb-unc-119(+)]*	This study
DLS817	*cul-6(ok1614) IV; skr-3(ok365) skr-5(rhd269) V; mgIs70[*P*vit-3::GFP]; rhdIs4[*P*vha-6::mCherry::his-58::SL2::fbxl-5 + cb-unc-119(+)]*	This study
DLS863	*uba-1(it129) IV; mgIs70[Pvit-3::GFP]; rhdIs4[*P*vha-6::mCherry::his-58::SL2::fbxl-5 + cb-unc-119(+)]*	This study
DLS874	*reSi5[*P*ges-1::TIR1::F2A::mTagBFP2::NLS::AID::tbb-2 3'UTR] I; rhdSi42[*P*vit-3::mCherry::unc-54 3'UTR + cb-unc-119(+)] II; fbxl-5(rhd298[3xFLAG::AID::fbxl-5]) V*	This study
DLS884	*rhdSi42[*P*vit-3::mCherry::unc-54 3'UTR + cb-unc-119(+)] lin-4(e912) II; fbxl-5(rhd304) V*	This study
DLS885	*rhdSi42[*P*vit-3::mCherry::unc-54 3'UTR + cb-unc-119(+)] lin-4(e912) II; fbxl-5(rhd305) V*	This study
DLS886	*rhdSi42[*P*vit-3::mCherry::unc-54 3'UTR + cb-unc-119(+)] lin-*	This study

	4(e912) II; fbxl-5(rhd306) V	
DLS889	*reSi5[Pges-1::TIR1::F2A::mTagBFP2::NLS::AID::tbb-2 3'UTR] I; rhdSi42[*P*vit-3::mCherry::unc-54 3'UTR + cb-unc-119(+)] lin-4(e912) II; fbxl-5(rhd298[3xFLAG::AID::fbxl-5]) V*	This study
DLS946	*rict-1 & pqn-32(rhd314) II; mgIs70[Pvit-3::GFP]*	This study
DLS948	*rict-1(mg360) II; fbxl-5(rhd43) V; mgIs70[Pvit-3::GFP]*	This study
DLS949	*rict-1 & pqn-32(rhd314) II; fbxl-5(rhd43) V; mgIs70[Pvit-3::GFP]*	This study
GR2122	*mgIs70[*P*vit-3::GFP]*	Dowen et al., 2016
GR2123	*lin-4(e912) II; mgIs70[*P*vit-3::GFP]*	Dowen et al., 2016
GR2125	*lin-29(n333) II; mgIs70[*P*vit-3::GFP]*	Dowen et al., 2016
GR2140	*sgk-1(ok538) X; mgIs70[*P*vit-3::GFP]*	Dowen et al., 2016
GR2146	*daf-2(e1370) III; mgIs70[*P*vit-3::GFP]/+*	Dowen et al., 2016
GR2147	*rict-1(mg360) II; mgIs70[*P*vit-3::GFP]*	Dowen et al., 2016
MT333	*lin-29(n333) II*	Ambros and Horvitz, 1984

Supplementary Table S1. *C. elegans* strains used in this study. The strain names, genotypes, and associated references are shown.

Target gene	Location in gene, crRNA guide number	crRNA sequence	Alleles	Genomic edit
skr-5	5’ end, rhd32<br>3’ end, rhd33	rhd32: UCUCAUAAAAAGGCCUGUAA<br>rhd33: GGGCAAAUUUGGUCUUGAAG	*rhd269*	682 bp deletion
skr-4	Internal, rhd53	rhd53: UCCUUGAGAAGAUUAUCACC	*rhd283*	W67*
fbxl-5	5’ end, rhd60	rhd60: UACCUUUCAAAUUUCCAGAA	*rhd298*	3xFLAG::AID
fbxl-5	5’ end, rhd60<br>3’ end, rhd12	rhd60: UACCUUUCAAAUUUCCAGAA<br>rhd12: UCCAAUUGGUCCACACUCUG	*rhd304*	2,474 bp deletion
fbxl-5	5’ end, rhd60<br>3’ end, rhd12	rhd60: UACCUUUCAAAUUUCCAGAA<br>rhd12: UCCAAUUGGUCCACACUCUG	*rhd305*	2,508 bp deletion
fbxl-5	5’ end, rhd60<br>3’ end, rhd12	rhd60: UACCUUUCAAAUUUCCAGAA<br>rhd12: UCCAAUUGGUCCACACUCUG	*rhd306*	deletion, unknown size
rict-1	5’ end, rhd9<br>3’ end, rhd40	rhd9: AAAUUUCAAUUUUCAGGCGA<br>rhd40: GAAAAUACUUAUAAAUGGAA	*rhd314*	18,298 bp deletion

Supplementary Table S2. The crRNAs used in this study. A list of the crRNA guides that were used in this study, including their target genes, their ribonucleotide sequences, and the alleles and genomic edits that were generated with each edit.

mRNA Target	Primer Sequence (5' to 3')	Reference
act-1	F: GCTGGACGTGATCTTACTGATTACC<br>R: GTAGCAGAGCTTCTCCTTGATGTC	Hoogewijs et al., 2008
vit-1	F: GAGGTTCGCTTTGACGGATA<br>R: GGCTTCACATTCCTCGTTCT	Ding and Grosshans, 2009
vit-2	F: GACACCGAGCTCATCCGCCCA<br>R: TTCCTTCTCTCCATTGACCT	DePina et al., 2011
vit-3/4/5	F: CATGTGCACCATCGAAGAACTC<br>R: CCAATGTGGTTTCAATGACAAGTTG	Dowen et al., 2016
vit-6	F: TTCACCCAGAAGCCAGTTC<br>R: AGGATGGGAGGCAGTAGAC	Dowen et al., 2016
fbxl-5	F: GCCAAACACAATCCAGTTCAG<br>R: AGAAGTCCGAAATCCAAGTCC	This study

Supplementary Table S3. The RT-qPCR primers. The sequences (5' to 3') of the qPCR primers used in this study, as well as any associated references.

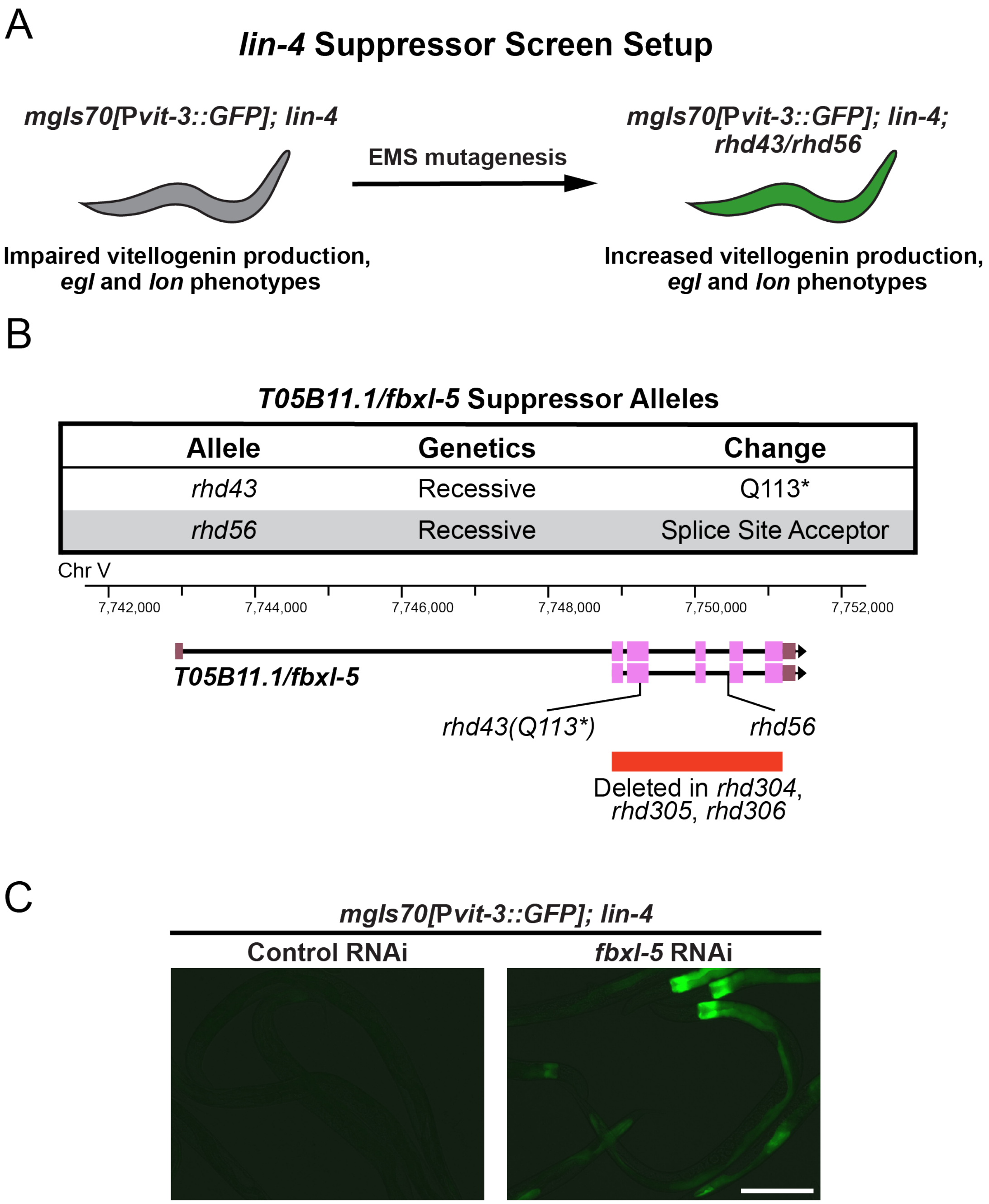

Supplementary Figure S1. Mutations in the *fbxl-5* gene partially suppress the vitellogenesis defects displayed by the *lin-4* mutant. (A) The design of the EMS mutagenesis screen employing the *mgIs70[*P*vit-3::GFP]* vitellogenesis reporter. The *lin-4(e912)* mutant fails to express the reporter, while the selected *lin-4* suppressor mutants express the reporter yet maintain the egg laying and long body defects (*egl* and *lon* phenotypes) that are associated with the *lin-4* mutation. This approach selects for mutations that impact hypodermal-to-intestine developmental signaling and selects against mutations that act cell-autonomously in the vulva or hypodermis to suppress *lin-4* (*i.e.*, *lin-14* mutations). **(B)** The two *fbxl-5* mutant alleles isolated in the *lin-4* suppressor screen (top) and a gene model illustrating where these mutations are positioned within the *fbxl-5* locus (bottom). The CRISPR-derived deletion mutations are also shown. **(C)** Representative fluorescence images of *mgIs70[*P*vit-3::GFP]* reporter expression in the *lin-4* mutant following control or *fbxl-5* RNAi (scale bar, 200 µm). The *mgIs70* transgene is an integrated high-copy transgene.

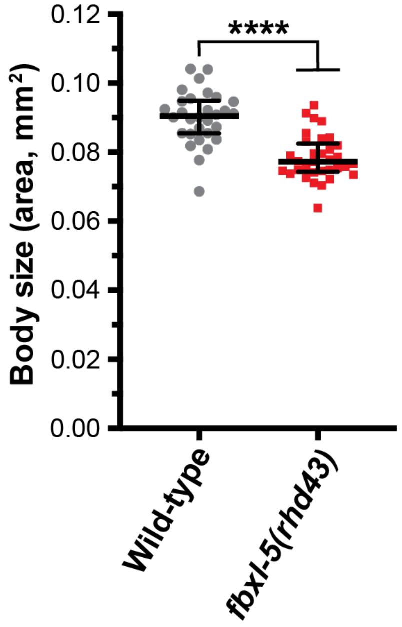

Supplementary Figure S2. Loss of *fbxl-5* reduces body size in wild-type animals. Body size measurements of wild-type and *fbxl-5(rhd43)* mutant animals (median and interquartile range; ****, $P<0.0001$, two-tailed T-test).

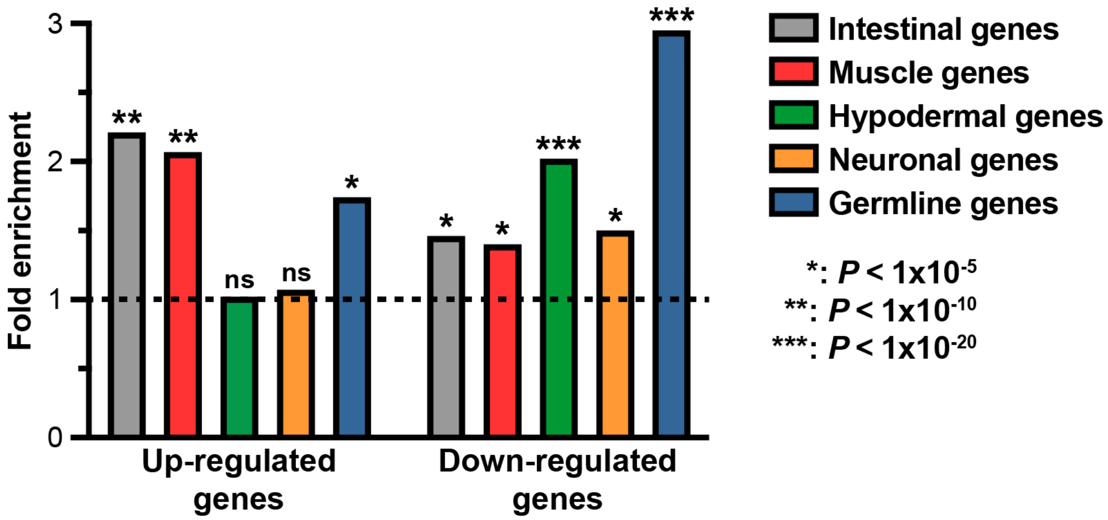

Supplementary Figure S3. Loss of *fbxl-5* impacts gene expression in several different tissues. Fold enrichment (observed/expected) for the differential expression of genes (mRNA-Seq of the *fbxl-5(rhd43)* mutant, 1% FDR) that are known to be expressed in the indicated tissues. The hypergeometric *P* values are reported.

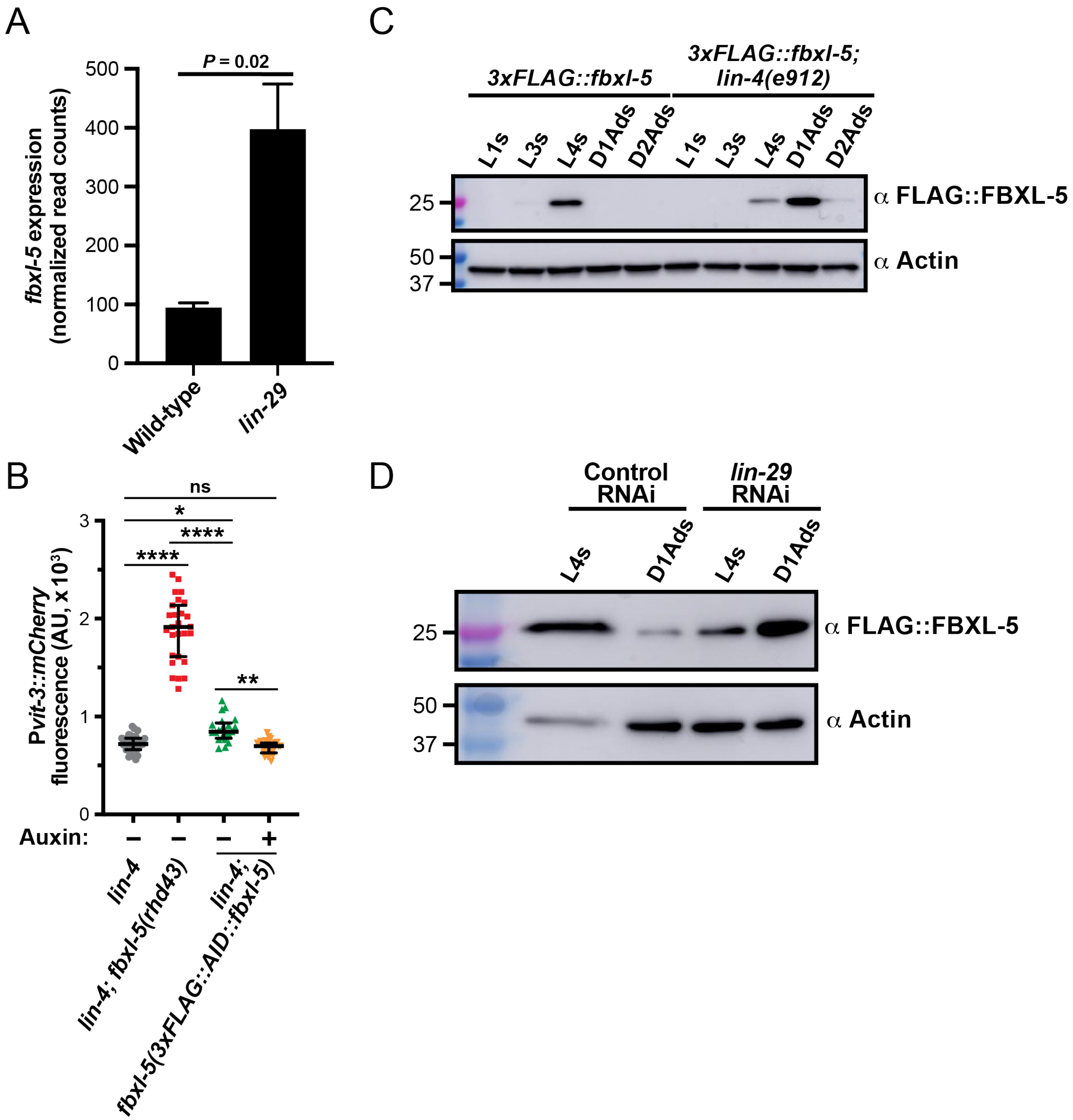

Supplementary Figure S4. The *fbxl-5* gene is ectopically expressed in adult *lin-4(e912)* and *lin-29(n333)* mutant animals. (A) Normalized mRNA-Seq read counts for the *fbxl-5* gene in wild-type or *lin-29(n333)* mutant animals at the day 1 adult stage (mean +/- SEM; two-tailed T-test). **(B)** Quantification of P*vit-3::mCherry* fluorescence in *lin-4(e912)*, *lin-4(e912); fbxl-5(rhd43),* or *lin-4(e912); fbxl-5(rhd298[3xFLAG::AID::fbxl-5])* day 1 adult animals. Auxin (4 mM) was applied to *lin-4(e912); fbxl-5(rhd298[3xFLAG::AID::fbxl-5])* animals at the L1 stage and maintained until adulthood. **(C)** A western blot analysis of lysates from wild-type or *lin-4* mutants expressing an endogenously-tagged 3xFLAG::FBXL-5 at the indicated developmental stages (replicate experiment shown in Figure 3B). An actin blot is included as a loading control. **(D)** A western blot analysis of lysates from L4 or day 1 adult wild-type animals expressing 3xFLAG::FBXL-5 protein after treatment with control or *lin-29* RNAi. An actin blot is included as a loading control.

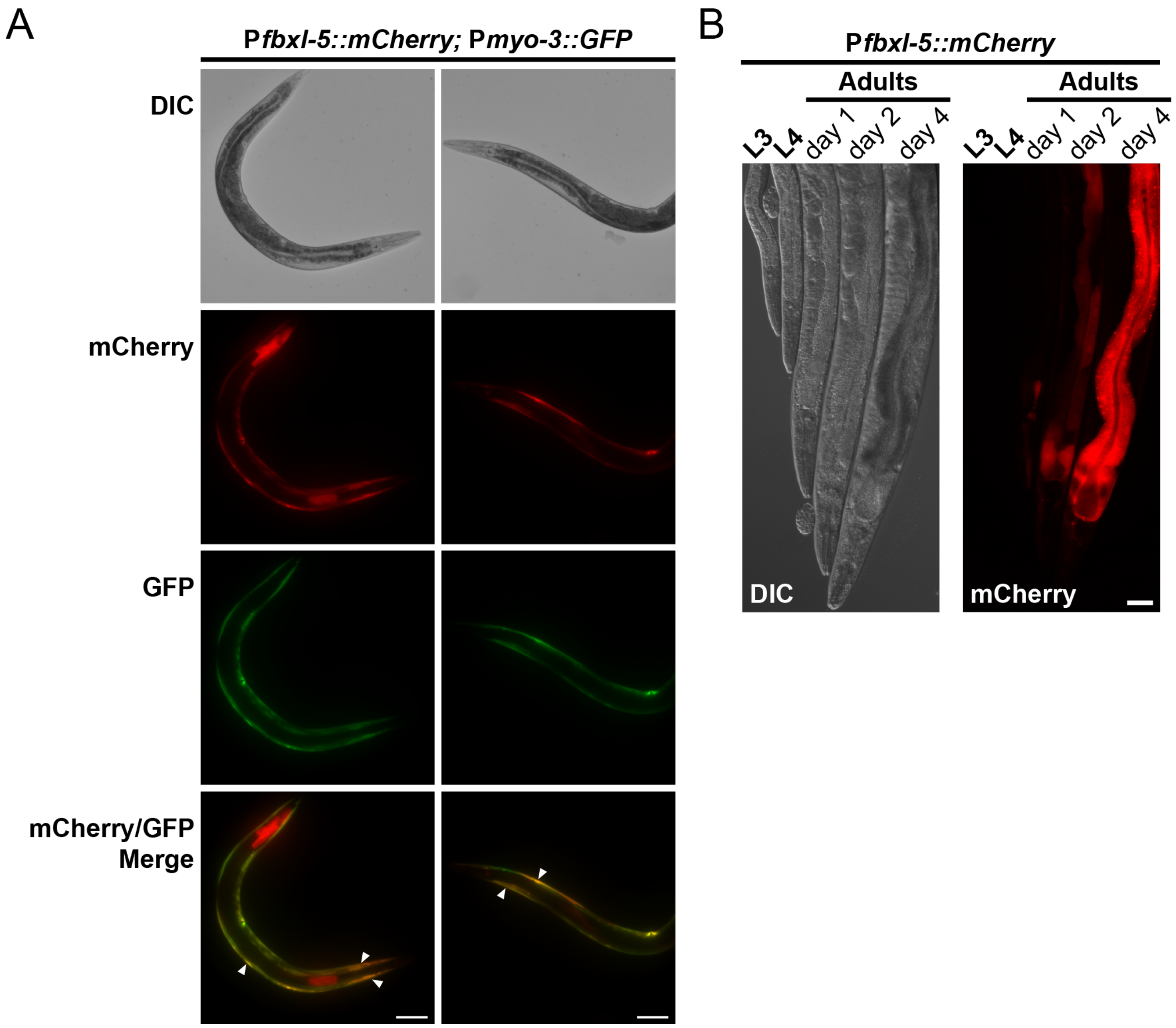

Supplementary Figure S5. Expression of *fbxl-5* in the body wall muscle and intestine. (A) Representative DIC, mCherry, GFP, and mCherry/GFP merged images of two individuals expressing transcriptional reporters for *fbxl-5* (P*fbxl-5::mCherry*) and *myo-3* (P*myo-3::GFP*), which marks the body wall muscle (scale bars, 100 µm). **(B)** Expression of P*fbxl-5::mCherry* in wild-type animals at the indicated developmental stages (scale bar, 100 µm). High levels of intestinal expression prevent visualization of mCherry expression in the body wall muscle.

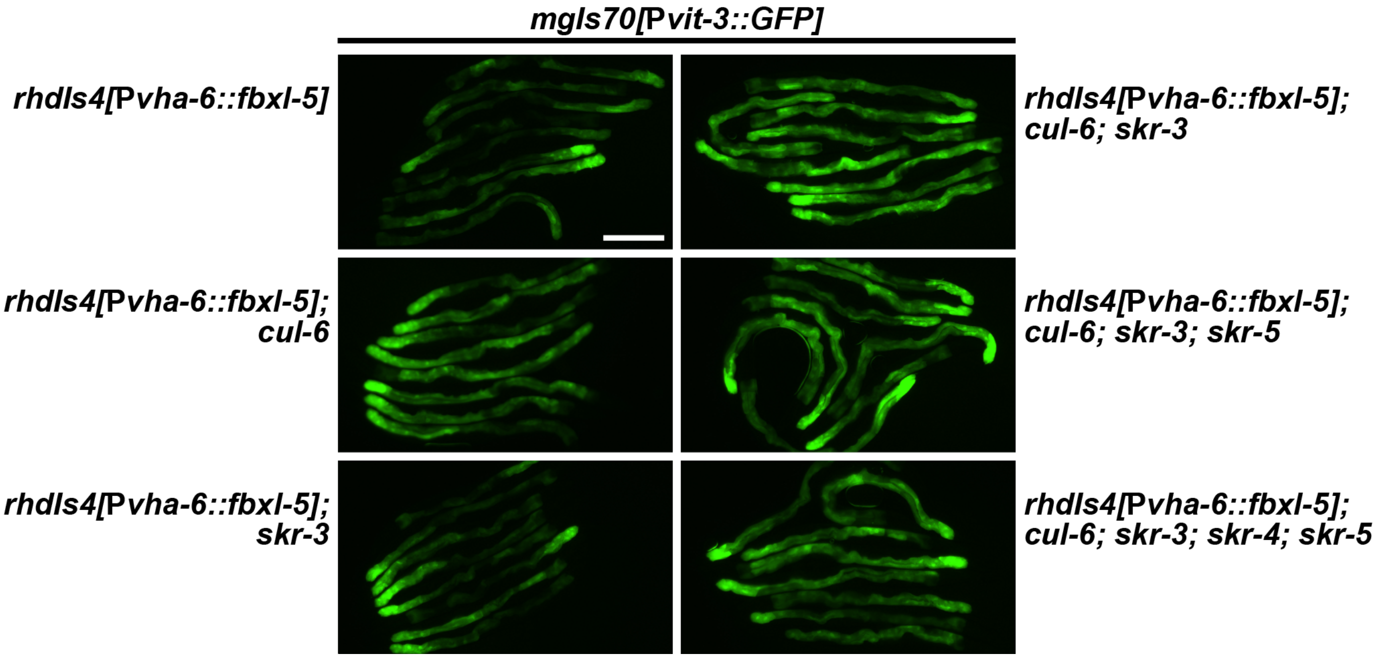

Supplementary Figure S6. FBXL-5-dependent repression of vitellogenesis requires *cul-6* and *skr-3*. Representative P*vit-3::GFP* fluorescence images of day 1 adult animals over-expressing *fbxl-5* in the indicated mutant backgrounds (scale bar, 200 μm). The same representative images for *rhdIs4* and *rhdIs4; cul-6; skr-3* can be found in Figure 6.

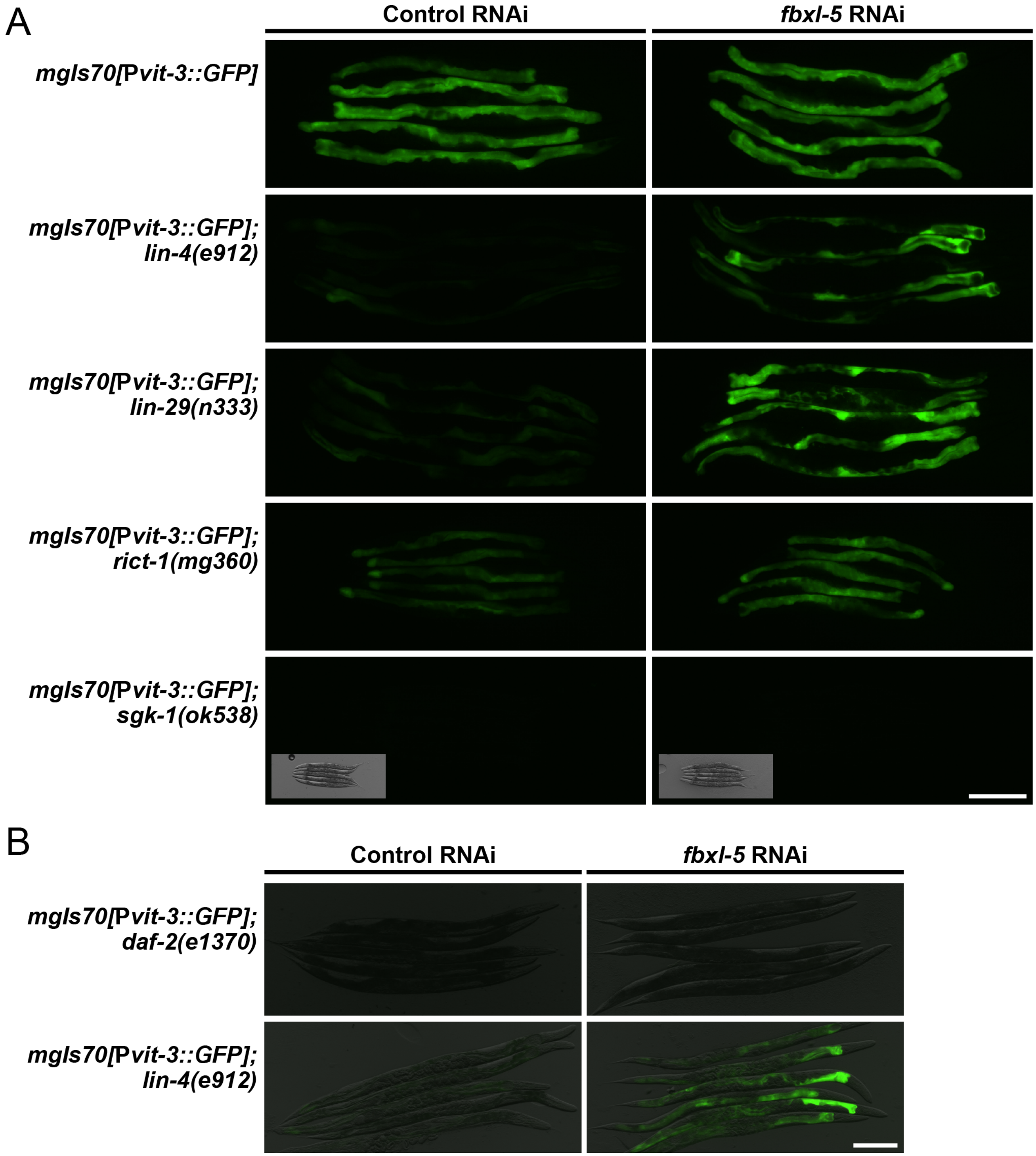

Supplementary Figure S7. Knock-down of *fbxl-5* suppresses mTORC2, but not insulin, mutants. (A, B) Representative P*vit-3::GFP* fluorescence images of the indicated mutants as day 1 adults following treatment with control or *fbxl-5* RNAi (scale bars, 200 μm). **(A)** The DIC images are included for the *sgk-1(ok538)* mutant since no GFP fluorescence is visible. **(B)** The GFP fluorescence images are overlaid on the DIC images.

References

Ambros, V., and Horvitz, H. R. (1984). Heterochronic mutants of the nematode Caenorhabditis elegans. *Science* 226, 409–416. doi: 10.1126/science.6494891.

Brenner, S. (1974). The genetics of Caenorhabditis elegans. *Genetics* 77, 71–94. doi: 10.1093/genetics/77.1.71.

DePina, A. S., Iser, W. B., Park, S.-S., Maudsley, S., Wilson, M. A., and Wolkow, C. A. (2011). Regulation of Caenorhabditis elegans vitellogenesis by DAF-2/IIS through separable transcriptional and posttranscriptional mechanisms. *BMC Physiol* 11, 11. doi: 10.1186/1472-6793-11-11.

Ding, X. C., and Grosshans, H. (2009). Repression of C. elegans microRNA targets at the initiation level of translation requires GW182 proteins. *EMBO J* 28, 213–222. doi: 10.1038/emboj.2008.275.

Dowen, R. H., Breen, P. C., Tullius, T., Conery, A. L., and Ruvkun, G. (2016). A microRNA program in the *C. elegans* hypodermis couples to intestinal mTORC2/PQM-1 signaling to modulate fat transport. *Genes Dev.* 30, 1515–1528. doi: 10.1101/gad.283895.116.

Hoogewijs, D., Houthoofd, K., Matthijssens, F., Vandesompele, J., and Vanfleteren, J. R. (2008). Selection and validation of a set of reliable reference genes for quantitative sod gene expression analysis in C. elegans. *BMC Mol Biol* 9, 9. doi: 10.1186/1471-2199-9-9.

Torzone, S. K., Park, A. Y., Breen, P. C., Cohen, N. R., and Dowen, R. H. (2023). Opposing action of the FLR-2 glycoprotein hormone and DRL-1/FLR-4 MAP kinases balance p38-mediated growth and lipid homeostasis in C. elegans. *PLoS Biol* 21, e3002320. doi: 10.1371/journal.pbio.3002320.
